# Physicians in private practice: reasons for being a social franchise member

**DOI:** 10.1186/1478-4505-10-25

**Published:** 2012-08-01

**Authors:** Dale Huntington, Gary Mundy, Nang Mo Hom, Qingfeng Li, Tin Aung

**Affiliations:** 1 Reproductive Health and Research Department, World Health Organization, Geneva, Switzerland; 2 Population Services international, 273 Kim Ma Street, Ba Dinh District, Ha Noi, Vietnam; 3 PSI/Myanmar, 16 West Shwe Gone Dine 4th Street,, Bahan Township, Yangon, Myanmar; 4 3101 Huntingdon Ave., Baltimore, MD, 21211, USA

**Keywords:** Social franchising, Reproductive health, Myanmar

## Abstract

**Background:**

Evidence is emerging on the cost-effectiveness, quality and health coverage of social franchises. But little is known about the motivations of providers to join or remain within a social franchise network, or the impact that franchise membership has on client volumes or revenue earnings.

**Methods:**

(i) Uncontrolled facility based of a random sample of 230 franchise members to assess self-reported motivations; (ii) A 24 month prospective cohort study of 3 cohorts of physicians who had been in the franchise for 4 years, 2 years and new members to track monthly case load and revenue generated.

**Results:**

The most common reasons for joining the franchise were access to high quality and cheap drugs (96.1%) and feelings of social responsibility, (95.2%). The effects of joining the franchise on the volume of family planning services is shown in the 2009 cohort where the average monthly service volume increased from 18.5 per physician to 70.6 per physician during their first 2 years in the franchise, (p<0.01). These gains are sustained during the 3^rd^ and 4^th^ year of franchise membership, as the 2007 cohort reported increases of monthly average family planning service volume from 71.2 per physician to 102.8 per physician (p<0.01). The net income of cohort 2009 increased significantly (p=0.024) during their first two years in the franchise. The results for cohorts 2007 and 2005 also show a generalized trend in increasing income.

**Conclusions:**

The findings show how franchise membership impacts the volume of franchise and non-franchised services. The increases in client volumes translated directly into increases in earnings among the franchise members, an unanticipated effect for providers who joined in order to better serve the poor. This finding has implications for the social franchise business model that relies upon subsidized medical products to reduce financial barriers for the poor. The increases in out of pocket payments for health care services that were not price controlled by the franchise is a concern. As the field of social franchises continues to mature its business models towards more sustainable and cost recovery management practices, attention should be given towards avoiding commercialization of services.

## Introduction

During the past decade social franchises have moved from an emergent, proof of concept stage of development to being established networks of private sector providers for reproductive health and other primary care services. At the close of 2011 there were 59 franchised networks of over 150,000 private practice providers spread over 35 low and middle income countries, serving an estimated 31 million poor patients annually [1]. There has been some variation in the types of providers who are members of a social franchise, but in general the following characteristics define a social franchise: clinics are operator owned, payments to the provider are fee for service (and are made by the patient, a third party, voucher or other system), services are quality controlled/standardized and include both franchise and non-franchise supported services, [2,3].

The goals of a social franchise network have remained remarkably consistent over this period of growth: (i) Access: increase coverage the number of providers and health care services offered; (ii) Cost-effectiveness: provide a service at an equal or lower cost to other service delivery options inclusive of subsidy or system costs; (iii) Quality: provide services that adhere to quality standards and improve the pre-existing level of quality; and (iv) Equity: serve all population groups, emphasizing those in need, [3,4]. The basic business model for the franchiser has also remained largely unchanged as well: the franchiser is dependent upon external funding to support the costs of network management, commodity subsidies, quality assurance. Some franchises have been moving towards more commercial models of operating the networks (e.g., charging membership fees), but none operate on a cost recovery basis.

The speed at which the private sector in general and social franchises in particular have expanded operations has outpaced the availability of evidence on their cost-effectiveness, changes in service quality, impact on health coverage, outcomes and equity, [5,6] Some evidence is emerging that indicates social franchised health services have had a positive impact on the number of repeat users of family planning, [7,8], service quality as perceived by clients [9], and the franchise's ability to serve poor and vulnerable populations [10] . But there has been insufficient attention in the published literature on either the motivations of providers to join or remain within a social franchise network, or to evaluate the impact that franchise membership has on client volumes or revenue earnings, [11]. With price capitations often set below the providers’ customary fees and the increased administrative burdens of franchised membership, the motivations of providers to join and remain within a social franchise are poorly understood. This information is critically important given recent evidence on relative high costs associated with managing a franchise [12]. However, there is in general very little evidence in the published literature on the sustainability of social franchise models, [13].

This study of providers in the Sun Quality Health network in Myanmar addresses this gap in the evidence base on social franchises through its exploration of provider motivations – both financial and non-monetary – for joining and remaining in a social franchise.

### Setting of the study

Population Services International/Myanmar established the Sun Quality Health (SQH) franchise in 2001 and by the end of 2011 the number of active members in the network reached 1,462. In late 2008 there were 748 physician - members of SQH who provided Reproductive Health (RH) services (out of a total of 797 SQH active members) spread over 140 townships in 12 states. Physicians are carefully selected to join the franchise through a subjective assessment conducted by SQH management, based on the provider's reputation, length of service, interest to services available in the network, the accessibility of the clinic to poor and the clinic conditions. As such, SQH members may be somewhat different than the general population of private practice physicians in Myanmar. Providers who join the SQH franchise are fee for service, licensed General Practitioners located in peri-urban areas of cities and small towns where multiple other sources of care are available, including government clinics. They work full-time in their clinics, many keeping their clinic open until 7 or 8 pm. Physicians are enrolled through a one-week long induction training, in batches of approximately 20. Annually around 100 new members have been added to the franchise network since its launch

Members of the SQH franchise provide both franchised supported (family planning, TB, pneumonia, malaria and HIV testing) and non-franchised services. For the franchise supported services, the provider agrees to a price capitation on the medical product (which is highly subsidized) and consultation fees. However, for non-franchise supported services there are no capitations. Franchise members also provide monthly reports on the volume of consultations and commodities sold for the franchise supported services and also agree to adhere to service quality standards including periodic quality control visits. In return the franchise members benefit by signage (that indicates quality standards to patients), received medical product at highly subsidized price, in-service training and up-to date information.

### Study design

The results from two separate study elements are reported on here. One element used an uncontrolled observational design: 230 Sun Quality Health clinics were randomly selected with probability proportionate to size based on average family planning case load (SQH statistical data source). From this sample of facilities, 228 member physicians agreed to be interviewed from 100 townships in 10 states and divisions spread across country.

The second element utilized a prospective cohort study design to examine changes in the providers' case load volume and income over a two year period. Based on anecdotal expert opinion from the SQH franchise management, the full effects of joining the franchise were estimated to become apparent only after a period of time had elapsed after the provider joined the franchise (in order to allow for the community served to recognize the benefits). Therefore a 2 year study period was used in the cohort study to allow for the more sustained effects to be evident, and to assess if different cohorts of franchise members are experiencing similar trajectories of revenue and case load growth. We selected 3 cohorts of providers to explore this supposed 2 year effect: providers who joined the franchise in 2005, 2007 and 2009 (i.e., those who had been in the franchise 4 years, 2 years or were new members at the time of our study).

### Sample characteristics

The majority (86.0%) of the providers in the facility-based survey were between 44 – 59 years, mean age was 52.5 years. Approximately two thirds were male (62.7%) and had been SQH members for 4 years or longer (69%). On average these providers have been working for 16.6 years at the clinic where the interview was carried out. The overall experience as General Practitioners was 24.1 years on average*.* Anecdotal evidence from SQH franchise management suggests that these characteristics are common with other members of the franchise, as recruitment targets older more established physicians of known quality.

All the physicians who joined the franchise network during each of the 3 selected years (2005, 2007 and 2009) were asked complete a specially developed form that reported monthly case load of all client types (franchise supported and not), net and gross income. Of those 266 providers we approached, 81.2% (n=216) agreed to take part in the study. The enrolled providers came from 78 towns located in 13 provinces of Myanmar. Data was collected between May 2009 and April 2011 (24 months).

The attrition rate was initially high (6.9%) during the first 6 months of data collection, (Table 1) which led us to introduce a monetary incentive of 15 US$ per quarter for all providers who returned a completed summaries. In addition, providers who were fully compliant for 3 consecutive months were put into a lottery that awarded 32 providers with different medical products (e.g., pediatrics stethoscopes, Glucometer, minor surgical kits). The combination of the monthly cash incentive and lottery prizes for sustained compliance had the effect of lowering the attrition rate to<2% for the remaining 18 months of the data collection period. There were no significant differences between the providers who agreed to take part in the prospective cohort study and other members of Sun Quality Health Franchise who were not in the study in terms of their gender, age and years practicing medicine – with the exception of the 2009 class which had slightly more women providers accept to take part in the cohort study, (p<.081).

**Table 1:** Cohort Study Sampling Results

**Year of Cohort**	**Total SQH membership in 2009**	**Total enrolled in sample**	**Total reporting/included in analysis**	**Attrition**	**Attrition Rate**
2005	76	58	52	9	15.5%
2007	87	76	75	4	5.3%
2009	103	82	77	13	15.9%
Total	266	216	204	26	12.0%

### Cohort study data analysis

The cohort study measured the monthly case load for 22 medical services that belong to 4 categories: Family Planning, Maternal Health, Child Health and Other. The data set contained a few missing values as some providers failed to report on all 22 services or income variables at some times during the 24 month reporting period. The final dataset used in our analysis contained 96,448 records of medical services, with a missing rate of 10% (n=11,264 missing values), a quite low missing rate for a 24-month prospective cohort study. Exploratory data analysis indicates that the relationship between service volume and time is generally linear, so linear interpolation method was used to impute the missing values.

Gross and net incomes are also reported by the participants in this study. The numbers of records of both incomes are 4,384, while they should be 4,896 in a perfectly balanced panel data. The missing rate is the same as that of medical services, 10%. Since the relationship between income and time also appeared to be linear, we imputed the missing values of gross and net income with linear methods as we did for the medical services data.

The longitudinal model we used in the analysis of the cohort study is formally expressed in the following way.

(1) $$Y_{\mathrm{it}}=U_{i}+\beta_{0}+\beta_{1}X_{\mathrm{it}}+\epsilon_{\mathrm{it}}$$

(2) $$U_{i}N\left(0,v^{2}\right)$$

(3) $$\epsilon_{\mathrm{it}}N\left(0,\tau^{2}\right)$$

This is a linear model with random effect where *Y*_*it*_ is the outcome variable reported by provider i at time t. *U*_*t*_ is provider-specific random effect, used to adjust for the unobserved heterogeneity among providers; for example, it’s reasonable to expect that some personality characteristics might affect service volumes and income above and beyond the franchise signage and products. The study didn’t measure personality traits so this variable is used in the model to reflect these types of unobserved effects that could lead to inconsistent model estimates (which are common in cross-sectional studies). *X*_*it*_ is the covariates of provider i at time t. Our study only collected time-invariant provider characteristics (e.g., gender, age at joining the franchise and date of joining the franchise). The interpretation of coefficients is similar to a cross-sectional model – the change in the outcome variable that is brought about by one unit change in covariates. The model diagnosis results didn’t favor the inclusion of more covariates because the sample size was relatively small and inclusion of more covariates significantly reduced the model’s statistical power in testing the significance of coefficients.

There are several approaches available to fit this model [14,15]. In this study, the number of providers is relatively small and the length of time is short; we could not find conclusive evidence supporting any particular correlation structure in the data. Therefore we used Generalized Estimation Equation (GEE) method because it can ensure the consistency of estimates even when the specified correlation structure is not exactly the true correlation structure, and because the number of providers is relatively small and the length of time interval is short, [16]. After we examined a series of relevant plots and estimates of Auto Correlation Function (ACF), we decided to use an autoregressive model of order 1 (AR1) correlation structure for gross and net incomes and all medical service variables, except one month injection which displays exchangeable (uniform) correlation structure.

## Results

The findings from the facility based study element revealed that the most common reasons given by physicians for joining the Sun Quality Health franchise were access to high quality and cheap drugs (96.1%) and feelings of social responsibility, i.e., helping the poor (95.2%). Older members (55–59 years of age) were significantly more likely to cite social responsibility (p=0.02) and having no perceived risk of decreased earnings (p=0.03) as motivating factors for joining the franchise. Other important considerations were being able to avail of training courses (87.7%) and opportunities for professional networking (55.7%) that came with franchise membership. Although in many social franchises members commonly report a sense of social responsibility, the strength of the sentiment in helping the poor as being a key motivation for being a member of a social franchise is somewhat unique to the Myanmar network, while the interest in training and access to drugs is common to most social franchises. Approximately one half (52.1%) of the SQH members reported that their earnings have increased as a result of joining the franchise, which they attributed to increased sales of the price-controlled injectable contraceptive – as well as general increase in patients for other reasons. This self-reported increase in revenue and case load is examined in more detail through the prospective cohort study.

### Cohort study element

In our analysis, the trajectories of the three cohorts of the SQH franchise are compared against the months since each joined the franchise. For example, because cohort 2009 joined the franchise 24 months later than cohort 2007 did, the performance of cohort 2009 in the 24^th^ month should be compared to the performance of 2007 in its 1^st^ month of reporting, i.e. both at their respective 24^th^ month in the franchise. Similarly the 24^th^ month of reporting for cohort 2007 is compared to the 1^st^ month of reporting for cohort 2005, i.e., both are at their respective 24^th^ month in the franchise.

**Figure 1:**
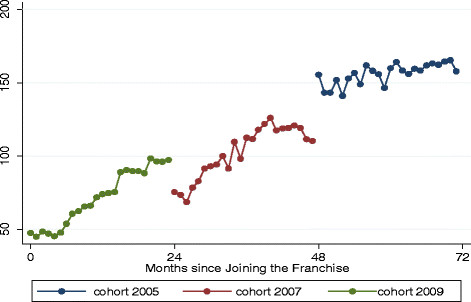
Number of Family Planning Consultations by Provider Cohort.

As shown in Figure 1, each of the three study cohorts experienced increases in the number of family planning consultations during the 24 months of data collection. The effects of joining the franchise on family planning services is clearly shown in the 2009 cohort where the average monthly service volume increased from 18.5 per physician to 70.6 per physician over the 24 month study period, (p <0.01). The increase during the first 24 months of membership in the franchise appears to be sustained during the second 24 month period as well, as shown in the finding that the 2007 cohort reported a significant increase of monthly average service volume from 71.2 per physician to 102.8 per physician (p<0.01). These effects appear to be diminished during the third 24 month period of franchise membership, as the 2005 cohort reported no significant change in family planning service volume. This pattern shows the accumulative positive impact of joining the social franchise on the medical service volume and income of providers. The Ministry of Health in Myanmar reported no increase in the use of family planning between 2009 – 2011 in the states where the cohort study was conducted,[17], although this data is at a high level of aggregation and is of limited value in making direct comparisons to the study sites. Prior to the study period the national fertility and reproductive health survey reported an increase in contraceptive prevalence. If that was the beginning of a trend it could suggest that the SQH providers in our study were benefiting by an overall increase in family planning use in the country.

Our longitudinal results of changes in medical services, summarized in Table 2, indicate that the longer the provider remains in the franchise, the greater likelihood that there will be increases in the case load of child health services (significant differences), and modest (although not statistically significant) increases in maternal and other types of service case loads. During the 24-month period when the data were reported, the average monthly volume of family planning services of cohort 2005 is 45.22 more than that of cohort 2007, and difference is statistically significant (p <0.05). Cohort 2007 also had a larger monthly volume of family planning services than cohort 2009 did. The same pattern is observed for child health services. Cohorts who joined the franchise earlier clearly were performing better with family planning services than cohorts joined later. However, for maternal health or other types of health services, the impact of joining the franchise is unclear. There is an important difference in the gender of the SQH providers: Women doctors performed better than their male counterparts in terms of both birth spacing methods and child health services. We conducted sensitivity analyses to investigate the potential effects that a single or small group of providers may be driving the cohort’s overall trajectory of change in family planning service volume (not shown in Table 2). Although there were 2 providers who exhibited large increases in family planning consultations, removing these cases from the analysis did not significantly change the regression coefficients.

**Table 2:** Longitudinal Model Results for 4 categories of health service monthly case load by cohort

Variable	**Birth Spacing Methods**		**Maternal Health Services**		**Child Health Services**		**Other Health Services**	
	**Cohort 2007 v.s. Cohort 2005**	**Cohort 2009 v.s. Cohort 2007**	**Cohort 2007 v.s. Cohort 2005**	**Cohort 2009 v.s. Cohort 2007**	**Cohort 2007 v.s. Cohort 2005**	**Cohort 2009 v.s. Cohort 2007**	**Cohort 2007 v.s. Cohort 2005**	**Cohort 2009 v.s. Cohort 2007**
Cohort	−45.22**	−33.00**	−39	16	−49***	−27**	−3	3
	(−87.09 - -3.36)	(−61.62 - -4.38)	(−112.22 - 34.32)	(−19.77 - 51.71)	(−81.98 - -15.18)	(−51.24 - -2.30)	(−8.45 - 2.56)	(−5.02 - 10.58)
Gender(reference: Male)	85.90***	37.02**	−19	−24	78***	34***	5	3
	(40.30 - 131.51)	(7.83 - 66.20)	(−98.69 - 60.94)	(−60.51 - 12.39)	(41.51 - 114.29)	(8.75 - 58.67)	(−1.17 - 10.83)	(−5.33 - 10.58)
Age at Joining Franchise	−3.77**	−0.45	−6**	−1	−4***	−1	−0	0
	(−6.86 - -0.68)	(−1.81 - 0.90)	(−10.95 - -0.15)	(−3.05 - 0.35)	(−6.28 - -1.36)	(−1.82 - 0.50)	(−0.61 - 0.21)	(−0.30 - 0.44)
Constant	314.39***	112.44***	442***	189***	317***	120***	27***	11
	(159.26 - 469.53)	(38.16 - 186.71)	(170.99 - 713.99)	(96.54 - 282.05)	(193.72 - 441.27)	(56.00 - 183.03)	(6.97 - 47.78)	(−9.36 - 31.13)
Observations	3,048	3,648	3,048	3,648	3,048	3,648	3,048	3,648
Number of Providers	127	152	127	152	127	152	127	152

Note: 95% confidence intervals in brackets, *** p<0.01, ** p<0.05, * p<0.1.

### Changes in income

**Figure 2:**
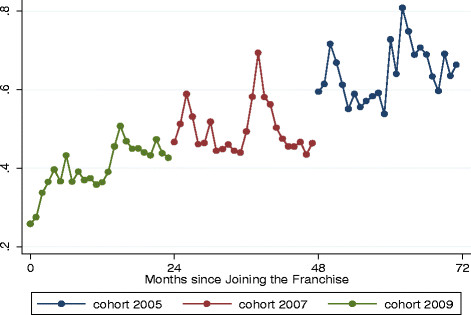
Trends in net income by study cohort.

The results in Figure 2 show that after 24 months the net income of cohort 2009 increased significantly (p=0.024) and reached approximately the same level as providers who had been in the franchise for 2 years (i.e., 2007). Furthermore, since the coefficient of time in the regression model is positive and statistically significant, we conclude that the income for the 2009 cohort is in an increasing trend despite the fluctuation (which is probably due to the effects of seasonal illnesses on clinic visits).

The results for cohort 2007 show that during the second and third year of franchise membership providers net income did not increase significantly; however, because the coefficient of time in the regression model is positive and statistically significant, we conclude that the monthly net income for this cohort is in a generalized increasing trend despite the fluctuations shown in the figure. The cohort 2005 exhibited the same pattern: no significant increase but a generalized trend towards larger net incomes. Overall, the three cohorts are on the different stages of the same trajectory in terms of net income growth, with short spikes that are probably associated with seasonal illnesses. The trends for gross income by cohort were essentially the same (not shown).

Sensitivity analyses were conducted to investigate the potential effects that a single or small group of providers may be driving the overall trajectory of change in net and gross income. Although there was 1 provider who exhibited a very large peak income in May 2010, there was no significant effect on the overall estimation of the model since dropping that provider only lead to an unnoticeable change to model results.

## Discussion

The SQH members reported a strong sense of social responsibility towards the poor and identified with the mission of PSI/Myanmar in ensuring equitable access to needed health services. Although not conclusive, the findings in this study are suggestive of how the Sun Quality Health franchise is expanding coverage of reproductive health services, notably family planning, through a growth in the number of clients being served by its member physicians. The timing of this study follows upon mixed evidence from early research that suggests contraceptive prevalence had increased, so perhaps the increase in family planning clients observed in the franchised clinics is part of a larger trend towards uptake in contraceptive use in Myanmar. Interestingly, there was a generalized increase in the case load of other, non-franchised services as well, particularly child health (e.g., seasonal illnesses). These spill-over effects from the “fractional franchise” model onto other types of health care services being provided by the SQH physicians are an indication of how the franchise’s quality improvement measures are increasing patient satisfaction among this select group of health care providers.

The increases in client volumes translated directly into increases in earnings among the SQH members in this study. Although the motivation for increasing income was not cited by the majority of providers as an important consideration for joining the franchise, older physicians were more likely to value that SQH membership posed no risk to their income. After joining the franchise, the income of the physicians was seen to increase in a steady trend through the first 6 years of membership – both for the franchised family planning services as well as non-franchised services. This finding has implications for franchise business model, suggesting that other tactics for reducing financial barriers for the poor should be developed. For example, the SQH franchise management could turn attention to developing their membership's business competencies, in addition providing subsidized medical products as a means for ensuring equitable access to reproductive health services. Cross subsidization, sliding scales and fee waivers might reasonably be introduced as clinic revenues continue to increase. Alternatively, the SQH franchise management may consider passing some of the franchise costs onto the members, through charging membership fees.

The increases in service volume and revenue are supported by out of pocket payments for health care services that were not price controlled by the franchise is a concern. As the field of social franchises continues to expand and mature its business models towards more sustainable and cost recovery management practices, attention should be given towards avoiding commercialization of services least the goal of serving the poor becomes thwarted by increased payments for other services. Integration into national social health insurance schemes, the use of vouchers and other types of demand side financing can be developed in Myanmar and other settings to lessen the out of pocket expenses for obtaining needed primary health care services by the poor.

## Competing interests

The authors declare that they have no competing interest.

## Authors’ contributions

DH conceived the study; DH, TA and NMH designed the study; NMH and LQ conducted the data analysis; all authors participated in the interpretation of the findings and prepared text for the paper. All authors read and approved the final manuscript.
